# The resection of extraosseous osteosarcoma was accompanied by the occurrence of pulmonary metastasis and distal metastasis at the primary site: a case report

**DOI:** 10.3389/fonc.2025.1549722

**Published:** 2025-04-16

**Authors:** Han Liu, Leilei Tian, Jianhua Mu, Xuanhong He, Li Zhuangzhuang, Taojun Gong, Jingjing Wang, Li Min, Minxun Lu, Chongqi Tu

**Affiliations:** ^1^ Department of Orthopedics, Orthopaedic Research Institute, West China Hospital, Sichuan University, Chengdu, China; ^2^ Department of Anesthesiology, West China Hospital, Sichuan University/West China School of Nursing, Sichuan University, Chengdu, China; ^3^ Department of Endocrine, Sichuan Provincial People’s Hospital, School of Medicine, University of Electronic Science and Technology of China, Chengdu, China

**Keywords:** extraskeletal osteosarcoma, ESOs, rare, maligancy, metastasis

## Abstract

**Background:**

Extraskeletal osteosarcoma (ESOS) is a rare and aggressive malignancy, comprising approximately 1% of all soft tissue sarcomas and 4.3% of all osteosarcomas. It predominantly affects individuals between the ages of 48 and 60, with a slightly higher incidence observed in males compared to females. The clinical diagnosis of ESOS poses a significant challenge due to its atypical presentation and overlap with other soft tissue neoplasms. Despite advances in diagnostic imaging and histopathological techniques, there is currently no consensus on the optimal treatment strategy.

**Case presentation:**

We report a case of a 64-year-old Chinese woman with ESOS of the left knee for 7 years, who experienced multiple recurrences after surgical resection, accompanied by systemic multiple soft tissue metastases and lung metastases. Initially, the patient found a painless mass in the left knee, which was diagnosed as a benign soft tissue mass at a local hospital and surgically removed. However, two years after the surgery, a mass recurred around the left knee and was larger than before, prompting the patient to seek treatment at our department. The patient underwent standard surgical treatment in our department, and postoperative histopathology, genetic testing, and immunohistochemical examination all confirmed the diagnosis of ESOS. Over the course of 5 years, the patient experienced multiple recurrences, and we attempted surgical treatment combined with chemotherapy and targeted therapy. Ultimately, due to multiple tumor ruptures in the left lower limb, severe pain, and sleep disorders, the patient decided to undergo left hip disarticulation surgery.

**Conclusion:**

The diagnosis and treatment of ESOS are challenging and require multimodal examination, including histological and immunohistochemical analysis. ESOS is rare, especially when it metastasizes to the distal soft tissues of the primary lesion site, which may portend a poorer clinical outcome compared to pure pulmonary metastasis. Despite current therapeutic interventions, this case still emphasizes its aggressiveness and poor prognosis.

## Introduction

According to the World Health Organization, ESOS is a malignant tumor characterized by the production of osteoid or bone matrix by neoplastic cells, arising without connection to the skeletal system (1). EOS is a high-grade mesenchymal soft tissue malignancy, accounting for approximately 1% of all soft tissue sarcomas and 2–4% of all osteosarcomas (2). By definition, EOS produces osteoid or cartilage matrix in a sarcomatous pattern and originates in soft tissue without attachment to adjacent osseous structures, with a slight male predominance (3–5). It typically arises in the deep soft tissues of the lower extremities (especially the thigh and buttocks), but has also been reported in various visceral organs (3). Radiologically, EOS presents as soft-tissue opacities with calcifications or osteoid matrix. Tumor enhancement is seen on CT or MRI following intravenous contrast, depending on the degree of necrosis. Unlike conventional osteosarcoma, which peaks during the second decade of life, EOS frequently develops in patients over 30 years old, with a peak incidence in the sixth decade (5, 6). Radiation or trauma is associated with up to 10% of cases (6). Metastases commonly affect the lungs, bones, lymph nodes, brain, liver, and skin. EOS is associated with a worse prognosis than conventional osteosarcoma, due to its higher rates of local recurrence and metastasis, especially to the lungs (7). Surgical resection of the primary tumor is the preferred treatment (7–10). For metastatic disease, doxorubicin-based chemotherapy and radiotherapy are commonly employed, although recent larger studies have questioned the efficacy of these treatments. The reported 5-year survival rate ranges from 12% to 77% (11).

To the best of our knowledge, no cases of ESOS metastasizing to the distal limb of the primary site have been reported in the literature. In this report, we present a case of ESOS with repeated recurrences and metastasis to the distal limb of the primary site.

## Case presentation

**Figure 1:**
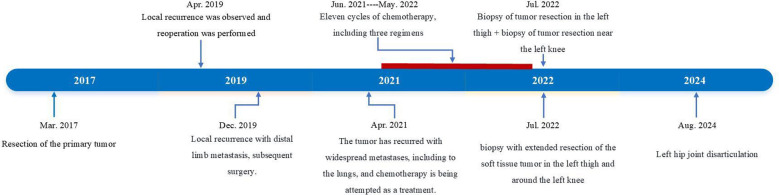
The timeline of the disease.

A 64-year-old woman presented with a seven-year history of extraskeletal osteosarcoma (ESOS) of the left knee (**Figure 1**). Seven years prior to admission, she noted a firm, asymptomatic mass on the lateral aspect of her left knee that did not affect her daily activities. There were no accompanying symptoms such as weakness, numbness, swelling, or discomfort in the left lower limb. At a local hospital, the mass was initially diagnosed as a benign soft tissue tumor, and the patient underwent surgical excision. However, the surgical wound exhibited poor healing, leading to localized scarring and recurrent fluid leakage. After multiple dressing changes, the incision eventually healed, and no additional treatments were administered. The patient was advised to attend regular follow-up appointments.

**Figure 2:**
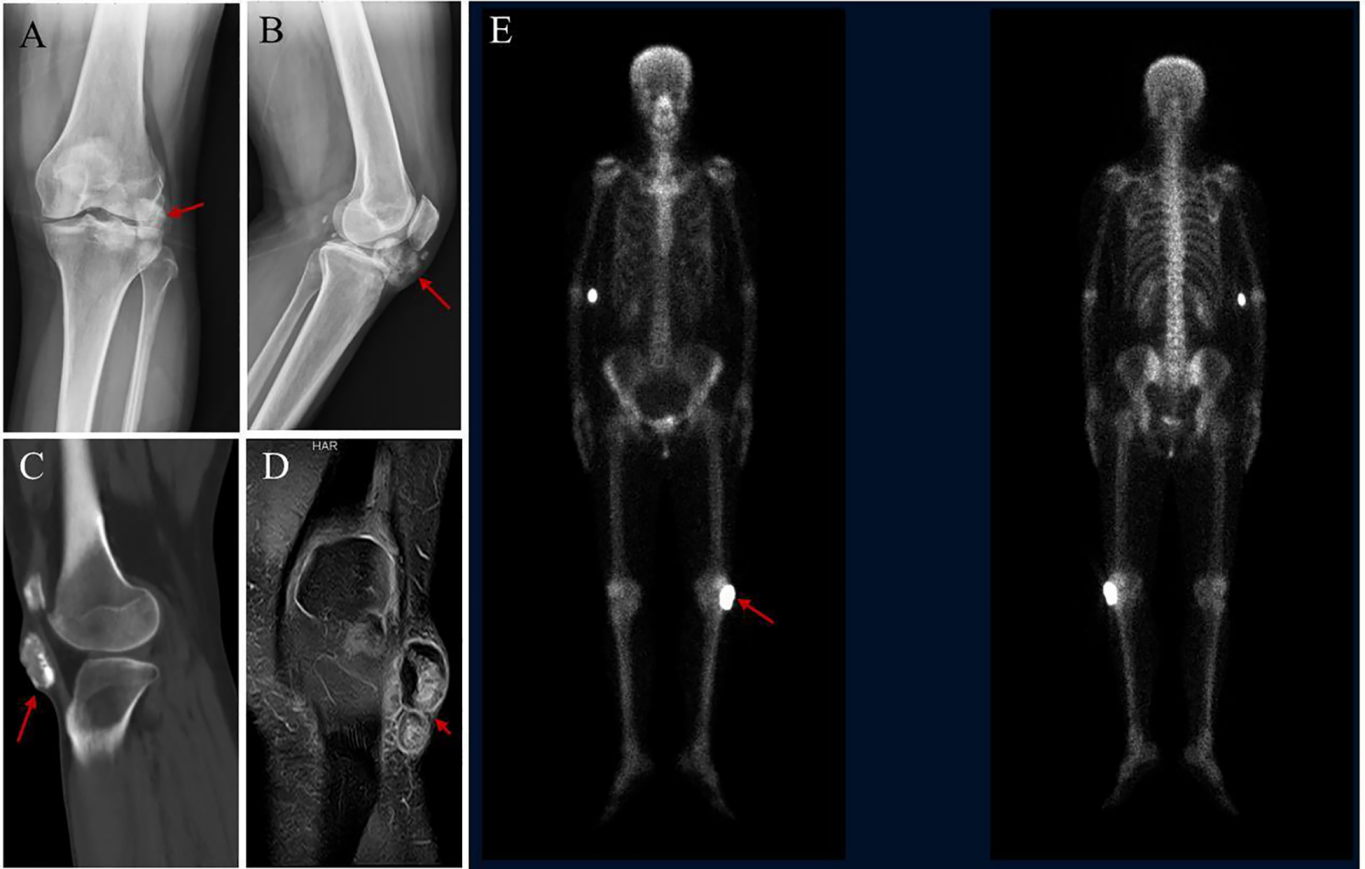
Radiological findings of the lesion. **(A, B)** X-ray shows nodular and flake-like high-density shadows under the left patella, approximately 5.4×4.5cm in size, with rough edges. **(C)** CT showed nodular high- density shadow in the subcutaneous tissue of the anterior inferior part of the left knee joint. **(D)** MRI showed a nodular short T2 signal shadow in the subcutaneous tissue of the anterior and inferior part of the left knee, with high signal intensity and low signal edge by fat suppression. **(E)** SPECT showed increased bone metabolism in the left knee joint region.

Five years ago, a similar lump reappeared at the same site, prompting a referral to our department. Imaging confirmed tumor recurrence (**Figures 2A-E**). The patient underwent an extensive surgical procedure on the left knee, including tumor resection biopsy, joint debridement, ligament reconstruction, pedicled fascial flap formation, skin grafting, and neurovascular exploration and decompression. Postoperative pathology revealed an osteosarcoma predominantly exhibiting osteoblastic features. Fluorescence *in situ* hybridization (FISH) testing showed no MDM2 amplification or USP6 translocation. Immunohistochemical results were as follows: SATB2 (partial +), SMA (+), CD34 (+), CDK4 (+/-), Desmin (-), S-100 (-), myogenin (-), and MDM2 (-), confirming the diagnosis of ESOS.

**Figure 3:**
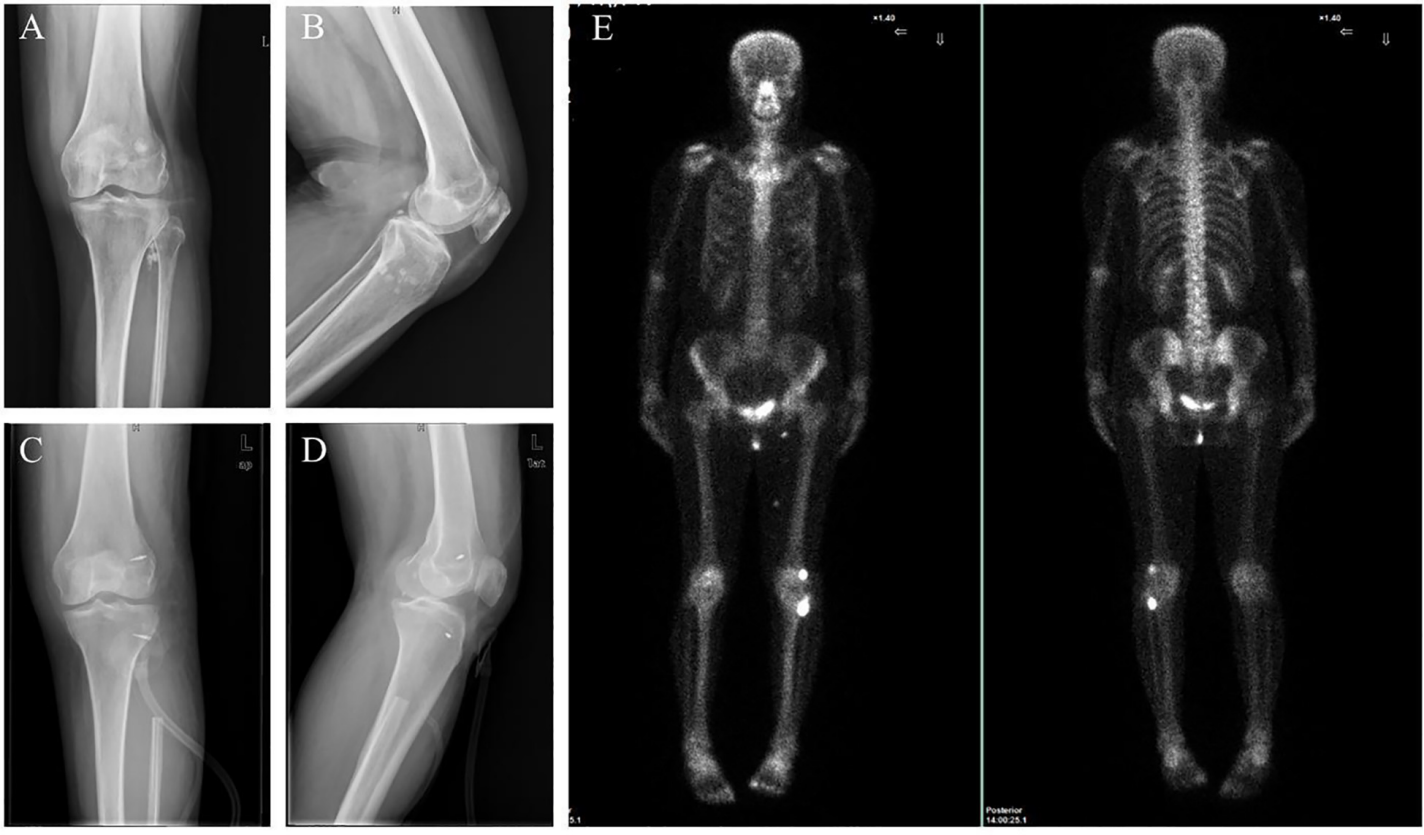
Imaging results indicate that the tumor has recurred. **(A, B)** X-ray reveals several small, dense nodules near the left knee joint. **(C, D)** Radiographs of the left knee after surgery. **(E)** SPECT showed increased bone metabolism in the left knee joint region.

Six months later, masses similar to those previously observed recurred both proximal and distal to the left knee joint. Imaging again confirmed tumor recurrence (**Figures 3A-E**). A second comprehensive surgical intervention was performed, involving removal and biopsy of tumors around the left knee joint and the proximal fibula, detailed vascular and nerve examinations, fascial reconstruction, restoration of the tibialis anterior muscle attachment, collateral ligament reconstruction, and Vacuum-Assisted Closure (VAC) therapy. Postoperative pathology demonstrated osteoblastic osteosarcoma-like changes in both the knee and proximal fibula regions, with infiltration into muscle and subcutaneous soft tissue. Margins were clear of tumor cells. Following surgery, the patient pursued traditional Chinese medicine treatment.

Three years ago, the patient noted multiple hard, painful masses emerging above and below the left knee joint, along the inner side of the upper and middle thigh, and near the lateral aspect of the upper thigh. She returned to our orthopedic department. A PET/CT scan revealed multiple nodules and masses in both lower limbs and in the left erector spinae muscles, with increased glucose metabolism. These lesions, located in subcutaneous or intermuscular tissues of the left hip, left thigh, around the left knee, the upper segment of the left lower leg, and the left erector spinae, were heterogeneous and showed signs of ossification. The largest mass, at the root of the left thigh, measured approximately 46 × 27 mm. Most lesions exhibited abnormal 18F-FDG uptake (SUVmax:7.44), strongly suggesting recurrent ESOS. Chemotherapy was recommended, and the patient underwent regular chemotherapy in our department.

Over the course of 11 cycles, three different chemotherapy regimens were administered. Between June 11, 2021, and October 25, 2021, the patient received five cycles of the AP regimen (liposomal doxorubicin 50 mg on day 1; cisplatin 60 mg on days 1-3, administered every three weeks). On November 24, 2021, and January 2, 2022, following a 20% dose reduction, two cycles of the IE regimen were administered (ifosfamide 2800 mg on days 1-5; etoposide 150 mg on days 1-5, every three weeks). From March 3, 2022, to May 24, 2022, four cycles of the GT regimen were given (gemcitabine 1400 mg on days 1 and 8; docetaxel 120 mg on day 8, every three weeks).

After the eighth cycle of chemotherapy, erythema and increased skin temperature developed over the left leg mass, followed by spontaneous ulceration and leakage of pale-yellow fluid or blood. These symptoms were managed with repeated dressing changes. Tumor progression was monitored periodically.

**Figure 4:**
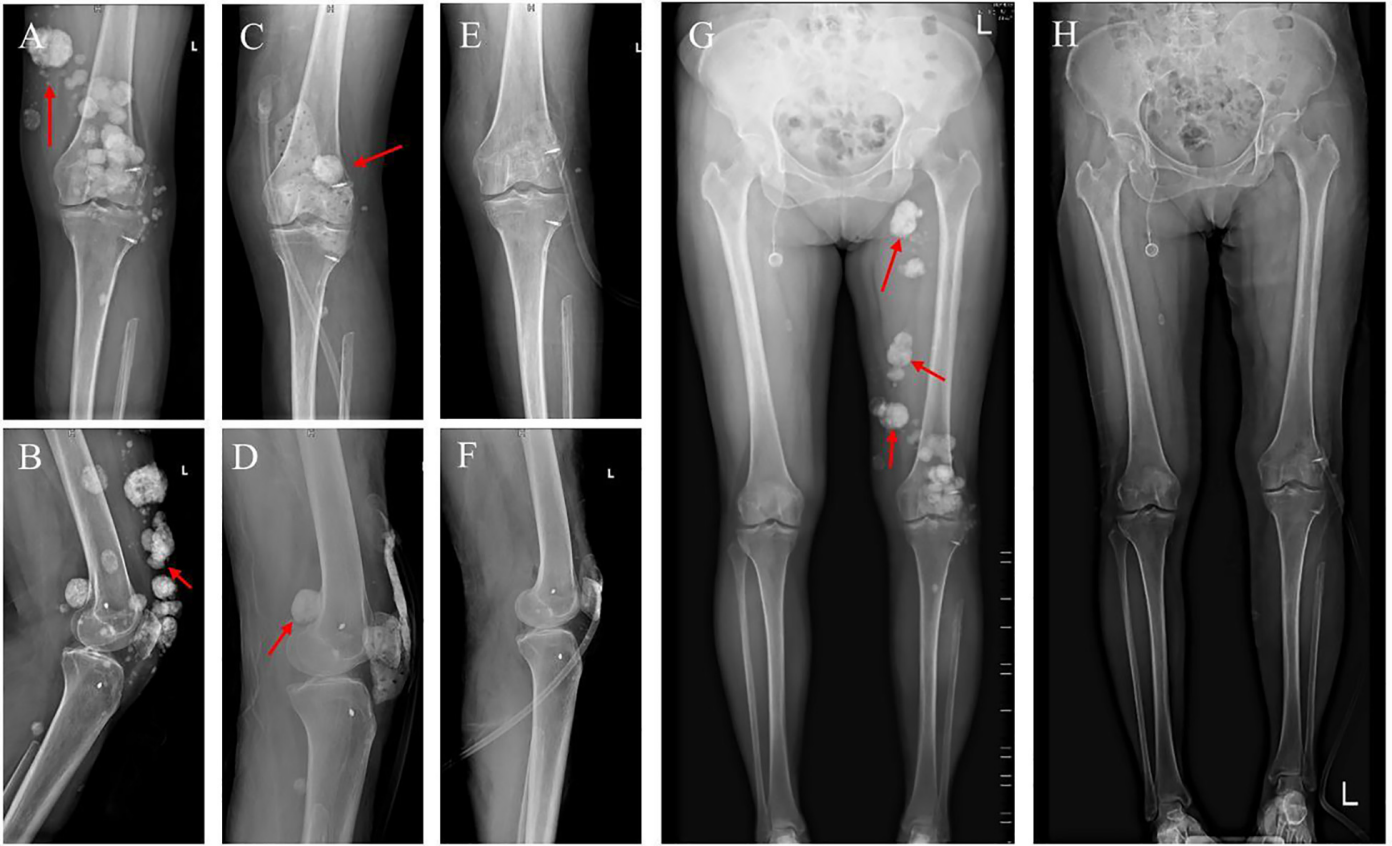
Imaging results before and after surgery. **(A, B)** Preoperative X-ray of the left knee joint. **(C, D)** Postoperative X-ray of the left knee joint. **(E, F)** X-ray after further surgery. **(G, H)** Full-length standing X-rays of both legs taken before surgery and after the extended resection procedure.

Two years ago, at a scheduled follow-up, X-ray imaging revealed multiple calcified nodules of varying sizes within the subcutaneous fat layer of the left buttock, hip, leg, around the left knee joint, in the posterior muscle compartments, and within the right thigh muscles (**Figures 4A, B, G**). On July 7, 2022, the patient underwent excisional biopsy of the left thigh tumor and peripatellar tumor, along with neurovascular exploration and decompression, local fascial flap formation, and VAC therapy.

Postoperative pathology revealed extensive irregular proliferation of trabecular bone-like tissue with significant degeneration and necrosis. A few atypical cells with deeply stained nuclei were noted, and in some areas, the tumor involved the superficial dermis and infiltrated sudoriferous glands. These findings were consistent with chemotherapy-treated osteosarcoma exhibiting osteoblastoma-like and sclerosing osteosarcoma-like changes. Based on these pathologic and imaging findings (**Figures 4C, D**), another procedure was performed on July 16, 2022, involving expansive debridement of the left thigh and peri-knee area, extended excisional biopsy of soft tissue tumors in these regions, harvesting a skin graft from the left thigh, skin grafting of the left lower limb, and VAC therapy (**Figures 4E, F, H**).

Postoperative pathology remained consistent with osteosarcoma changes after chemotherapy, featuring degenerative necrosis and irregular hyperplasia of trabecular bone. After wound healing, multiple new masses appeared in the left thigh and lower leg, as well as in the left inguinal region. The patient subsequently completed six additional cycles of chemotherapy.

In March 2024, the patient began taking targeted therapies (Apatinib Mesylate Tablets and Anlotinib). Despite this, multiple masses around the left knee ulcerated and drained fluid. Repeated dressing changes did not achieve complete wound healing, and the ulcerated areas gradually expanded. The patient’s pain intensified, necessitating long-term Tramadol, although its analgesic effect was limited, resulting in chronic insomnia. After extensive discussions, the patient elected to undergo amputation.

Physical examination and imaging confirmed the severity of her condition (**Supplementary Figures 5A-C, G-J, K**). In August 2024, the patient underwent left hip disarticulation with iliac fossa and inguinal lymph node dissection, as well as myofascial flap transposition plasty (**Supplementary Figures 5D-F**). Postoperative pathology revealed extensive proliferation of woven bone-like tissue with areas of high-grade osteosarcoma and osteoid matrix formation, consistent with recurrent osteosarcoma changes following chemotherapy.

## Discussion

ESOS is a high-grade spindle cell tumor, typically larger than 5 cm. Jenson et al. reported that 18 out of 25 cases were intramuscular (12). Patients with ESOS often present with a slowly growing soft tissue mass, with symptom duration ranging from 4 to 6 months (12, 13). Approximately 50% of patients experience pain in the area of the lesion (13). A history of radiation exposure and trauma are associated with the development of ESOS, with prior radiation exposure noted in about 10% of cases. Laskin et al. reported that 13% of all sarcoma cases are radiation-related ESOS. Trauma history is present in 12–30% of cases and is essential for differential diagnosis, as it may lead to conditions like myositis ossificans, which can mimic ESOS (12, 13). Notably, osteosarcoma development from myositis has been documented in the literature.

Proper history-taking and physical examination are critical in managing tumor lesions; however, radiological evaluation is indispensable for diagnosis. Initial imaging studies, such as plain radiography, may show soft tissue tumors with coarse or punctate calcifications. Magnetic resonance imaging (MRI) is crucial for local staging and preoperative planning, while computed tomography (CT) helps identify mineralization, necrosis, bone involvement, and metastases. Bone scintigraphy and positron emission tomography (PET) scans assist in staging the tumor (13, 14).

Malignant tumors are often nonspecific or asymptomatic in early stages, complicating diagnosis. Surgeons must consider alternative diagnoses beyond clinical judgment. Post-surgical analysis, including pathological, immunohistochemical, and genetic testing, is essential. In this case, testing revealed diffuse spindle cell proliferation with extensive osteochondral metaplasia, actively proliferating cells with large nucleoli, and nuclear division, suggesting osteosarcoma. The diagnostic overlap between ESOS and other diseases, both radiologically and pathologically, poses challenges. Accurate diagnosis is crucial, as ESOS requires more aggressive treatment strategies than other sarcomas (10, 15).

There are no definitive immunohistochemical (IHC) markers for ESOS, but IHC can aid diagnosis and exclude other possibilities. Osteoblastic ESOS may express SATB2, though this is not specific to ESOS, as other tumors producing bone can also express SATB2 (16, 17). In some cases, ESOS may weakly express SMA, S-100, and cytokeratins (18). Genetic findings in ESOS are nonspecific; approximately 10–20% of cases exhibit MDM2 and CDK4 amplification, typically seen in dedifferentiated liposarcoma (17, 19, 20). H3K27me3 deletion, more common in malignant peripheral nerve sheath tumors (MPNST) with bone involvement, may occur in ESOS, and SOX10 expression should be checked to rule out MPNST (16, 17, 21).

In this case, SATB2 was positive, a common finding in ESOS (21). MPNST was excluded due to the absence of S-100 expression (17, 22). Other differential diagnoses, such as dedifferentiated liposarcoma and fibrosarcoma, were ruled out based on the lack of lipid components and fibroblast proliferation in hematoxylin–eosin staining. Given the typical histopathological features of osteosarcoma and the exclusion of other possibilities through IHC, a final diagnosis of ESOS was made.

ESOS is associated with poor morbidity and mortality due to the lack of standardized treatment guidelines. The optimal therapeutic approach remains unclear, as few cases have been reported. Some clinicians recommend treating ESOS like high-risk soft tissue sarcomas, while others prefer conventional osteosarcoma treatment protocols. Paludo et al. reported a 27% objective response rate to preoperative platinum-based chemotherapy in ESOS, though there was no significant survival advantage compared to non-platinum regimens (10). Older cohort studies reported a 5-year overall survival rate of 25% versus 66% with and without chemotherapy, respectively, suggesting chemotherapy might improve survival. However, Nystrom et al. found that the 5-year overall survival rate was only 11.7%, with chemotherapy slightly extending survival (16.4 months vs. 9.3 months, p = 0.16) (11, 23, 24).

Treatment strategies for ESOS depend on clinical judgment (14, 25). Most follow guidelines for soft tissue sarcomas (using anthracycline with or without ifosfamide) or primary bone sarcomas (with cisplatin, doxorubicin, ifosfamide, and methotrexate) (13). However, adjuvant multi-agent chemotherapy has not shown statistically significant improvements in disease-free survival and is not recommended for routine use (9, 13, 26, 27).

This case of a middle-aged woman with no metastasis at diagnosis, tumor recurrence 7 months after radical resection, and metastatic progression 24 months later, mirrors previous reports. Recurrence rates for ESOS range from 45% to 50% within 6 to 9 months post-surgery (11).

In exploring the mechanism of tumor metastasis, venous blood return plays a crucial role. Tumor cells spread from the primary tumor site to distant organs through the circulatory system, achieving metastasis. Hematogenous metastasis, as the main pathway for tumor cells to colonize distantly, involves tumor cells penetrating through vessel walls and using the bloodstream to reach distant organs. Additionally, the lymphatic system, as an important auxiliary part of the circulatory system, has its lymphangiogenic signaling pathways continuously activated, which not only inhibits the normalization of lymphatic function but also enhances tumor interstitial pressure, thereby affecting the penetration of drugs into tumor tissues and weakening their inhibitory effects on tumor cell proliferation and invasion (28–31).

Special mention should be made of the Batson venous plexus, a venous network connecting multiple internal organs with the vertebral venous plexus, which provides a potential pathway for tumor cell metastasis. Although the presence of metastatic lesions in the patient’s lungs suggests a possible hematogenous metastasis mechanism, the absence of symmetrical metastatic lesions in the right lower limb reduces this possibility. However, the possibility of some unknown special metastatic mechanism leading to distal metastasis from the primary site cannot be ruled out (32–36).

Multicentric osteosarcoma (MOS), as a rare subtype of osteosarcoma, accounts for only 1% to 5% of all osteosarcoma cases. This disease type is characterized by multiple non-metastatic osteosarcoma lesions in the patient’s systemic skeleton, with its uniqueness lying in the absence of visceral metastasis. Depending on the temporal sequence of lesion appearance, MOS can be further subdivided into synchronous and metachronous categories. The former refers to the occurrence of multiple independent primary lesions at the same time point, while the latter refers to the appearance of new non-metastatic tumor lesions after the primary lesion. In this study, during the follow-up period, the patient experienced the emergence of tumor lesions outside the primary site multiple times, even including rare distal lesions, suggesting that these distal lesions may not be due to metastasis, but rather a typical manifestation of MOS (37–42).

In summary, the distal metastasis in this patient may be the result of the combined action of multiple metastatic mechanisms. It is these complex metastatic mechanisms that increase the difficulty of diagnosing and treating extramedullary osteosarcoma. Based on this, we share our experience in diagnosis and treatment, aiming to optimize the diagnostic and therapeutic strategies for extramedullary osteosarcoma and improve the prognosis of patients.

## Conclusion

This case report highlights the complexities of diagnosing extraskeletal osteosarcoma (ESOS), emphasizing the need for a comprehensive multimodal evaluation. Histological analysis plays a pivotal role in diagnosing ESOS by revealing high-grade pleomorphic spindle cells and osteoid matrix involvement. Given the rarity of ESOS and its overlapping features with other conditions, immunohistochemistry (IHC) is critical for establishing a definitive diagnosis. Specifically, SATB2 positivity strongly supports the diagnosis of ESOS, while S-100 negativity helps exclude other differential diagnoses. Treatment strategies often mirror those for high-risk soft tissue sarcomas or conventional osteosarcomas, although various chemotherapy regimens have shown limited impact on overall survival. In this case, the patient experienced recurrence and metastasis, highlighting the aggressive behavior and poor prognosis of ESOS. This report provides valuable insights into the diagnostic process and therapeutic approach for ESOS, contributing to the refinement of its precise diagnosis and management.

## Data Availability

The original contributions presented in the study are included in the article/**Supplementary Material**. Further inquiries can be directed to the corresponding author.
