# Supplementary figures and images for: The resection of extraosseous osteosarcoma was accompanied by the occurrence of pulmonary metastasis and distal metastasis at the primary site: a case report
Source: Front Oncol. 2025 Apr 16;15:1549722. doi: 10.3389/fonc.2025.1549722 (PMC12040817; doi:10.3389/fonc.2025.1549722)

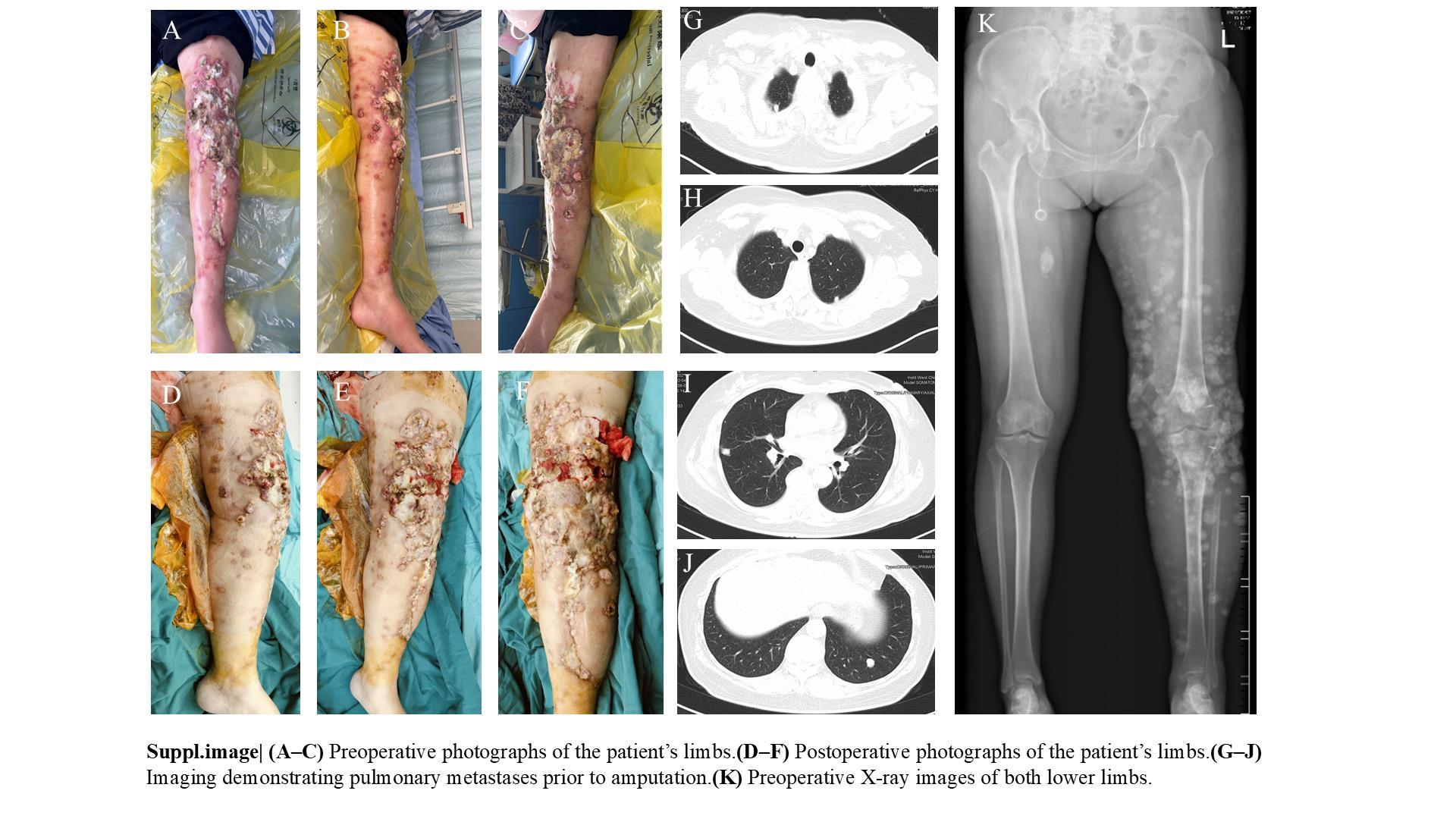

Supplement: Supplementary file 1 [file Image1.jpeg]
